# Xihuang Pill Induces Apoptosis of Human Glioblastoma U-87 MG Cells via Targeting ROS-Mediated Akt/mTOR/FOXO1 Pathway

**DOI:** 10.1155/2018/6049498

**Published:** 2018-06-26

**Authors:** Meng Shao, Zhenqiang He, Zhixin Yin, Peihong Ma, Qian Xiao, Yafeng Song, Ziming Huang, Yujie Ma, Yuqin Qiu, Aizhi Zhao, Taicheng Zhou, Qirui Wang

**Affiliations:** ^1^Department of Molecular Biology, State Administration of Traditional Chinese Medicine of the People's Republic of China, School of Traditional Chinese Medicine, Southern Medical University, Guangzhou, Guangdong 510515, China; ^2^Department of Neurosurgery/Neuro-Oncology, Sun Yat-sen University Cancer Center, State Key Laboratory of Oncology in South China, Collaborative Innovation Center for Cancer Medicine, Guangzhou, Guangdong 510060, China; ^3^Abramson Cancer Center, University of Pennsylvania Pereman School of Medicine, Philadelphia, PA 19104, USA; ^4^Department of Pharmacology, Yale School of Medicine, New Haven, CT 06520, USA; ^5^Department of Gastroenterological Surgery and Hernia Center, The Sixth Affiliated Hospital of Sun Yat-sen University, Guangzhou, Guangdong 510655, China

## Abstract

Xihuang pill (XHP), a traditional Chinese herbal formula, has long been used as an effective agent against multiple tumors. The aim of this study is to evaluate the effects of XHP on the growth inhibition and apoptosis in glioblastoma U-87 MG cells. Gas chromatography-mass spectrometry (GC-MS) was performed for constituent analysis of XHP. Cell viability, cell cycle arrest, generation of reactive oxygen species (ROS), and apoptosis were measured by CCK-8 assay, PI/RNase staining, DCFH-DA assay, TUNEL assay, Annexin V-FITC/PI double staining, and JC-1 assay, respectively. The role of XHP in the regulation of Akt/mTOR/FOXO1 interaction was clarified by using Western Blotting (WB), immunofluorescence (IF), pharmacological inhibitor or antioxidant, and siRNA silencing. The results suggested that XHP could inhibit U-87 MG cells growth and arrest cells in S-phase cell cycle significantly and that the generation of ROS, collapse of mitochondrial membrane potential, enhancement of Bax/Bcl-xL ratio, and reduction of the precursor forms of caspase-9 and caspase-3 caused by XHP prompted that a ROS-mediated mitochondria-dependent apoptosis was possibly involved. Furthermore, XHP affected the Akt/mTOR/FOXO1 pathway via inhibiting the phosphorylation of Akt, mTOR, and FOXO1 and increasing both prototype and nuclear translocation of FOXO1. Inhibition of Akt, mTOR, and FOXO1 by specific inhibitors or siRNA could interpose the apoptotic induction. In conclusion, we demonstrate for the first time that XHP may regulate glioblastoma U-87 MG cell apoptosis via ROS-mediated Akt/mTOR/FOXO1 pathway.

## 1. Introduction

Gliomas, a type of highly heterogeneous tumor, have been the leading lethal causes in primary central nerve system (CNS) tumors. According to the 2016 World Health Organization (WHO) classification of CNS tumors, gliomas in adults are mainly included astrocytoma (Grades I-IV), oligodendroglioma (Grades II-III), oligoastrocytoma (Grades II-III), and glioblastoma multiforme (GBM) (Grade IV) [1, 2]. GBM, the most aggressive and malignant form of gliomas, is distinguished by highly invasive behaviors with tentacle-like projections, making complete surgical removal difficult. Median overall survival of GBM patients is less than 18 months despite the adoption of optimized therapeutic scheme in combination of neurosurgery, chemotherapy, and radiotherapy [3]. In consideration of the significant toxicity and chemotherapy resistance in treatment, it is necessary to develop other effective strategies, for example, the exploration of potential benefits from traditional herbal formulas for glioblastoma treatment.

Xihuang pill (XHP, also called Xihuang Wan), a formula composed by four traditional Chinese medicines,* Olibanum*,* Myrrh*,* Moschus*, and* Calculus bovis*, has been used as a complementary and alternative medicine for tumor treatment in China since 18th century [4]. In modern clinical application, XHP has been proved effectively on various solid tumors including breast cancer [5], cervical cancer [6], glioma [7], colorectal cancer [8], and non-Hodgkin's lymphoma [9]. The experimental research showed that its ability to induce apoptosis in cancer cells or cancer stem cells (CSCs) was possibly attributed to the mitochondrial-related B-cell lymphoma- (Bcl-) 2 regulation [10, 11], extracellular signal-regulated kinase (ERK)/mitogen-activated protein kinase (MAPK) [12], or Wnt [13] signaling pathways, as well as cell cycle arrest [14, 15]. The invasion and metastasis inhibition were relevant to epithelial-mesenchymal transition (EMT) factor, Zinc finger E-box-binding homeobox 1 (ZEB1), and its downstream target genes ERK1/2, E-Cadherin, Occludin, matrix metalloprotein- (MMP-) 2, MMP-9, and junctional adhesion molecule- (JAM-) 1 [16, 17]. In addition, XHP could inhibit vasculogenic mimicry formation to reduce angiogenesis by downregulating expression of VE-Cadherin, MMP-2, and phosphorylated eph-receptor tyrosine kinase-type A2 (p-EphA2) [18]. Moreover, XHP exhibited the capability of converting tumor immunosuppressive microenvironment by reducing proportion of immunosuppressive cells such as myeloid-derived suppressor cells (MDSCs) and Treg cells [19], or by downregulating expression of cytokines such as interleukin- (IL-) 6, IL-10, and transforming growth factor- (TGF-) *β* [20] or by increasing IL-2, Interferon- (IFN-) *γ*, and the ratio of CD4^+^/CD8^+^ T cells [21, 22].

Despite the above widely described antitumor properties of XHP, little research has been devoted to GBM. On account of XHP's definite clinical efficacy on GBM and its unclear molecular mechanisms, additional studies on this subject are necessary. In this study, we investigated the effects of XHP on human U-87 MG glioblastoma cell growth and its underlying mechanisms related to ROS-mediated Akt/mTOR/FOXO1 pathway, so as to provide some experimental data to the further research and development on antiglioblastoma application of XHP.

## 2. Materials and Methods

### 2.1. Reagents and Antibodies

XHP was purchased from Tong Ren Tang Technologies Co., Ltd. (Beijing, China). Cell counting kit- (CCK-) 8 assay kit was obtained from Dojindo (Tokyo, Japan). PI/RNase Staining Buffer (RUO) for cell cycle detection was purchased from BD Biosciences (Franklin Lakes, USA). Annexin V-FITC/PI apoptosis kit (640914) was purchased from Biolegend (San Diego, USA). Reactive oxygen species assay kit, TdT-mediated dUTP nick end labeling (TUNEL) kit, and JC-1 kit were purchased from Beyotime Biotechnology (Beijing, China). LY294002 and rapamycin were purchased from MedChemExpress (Shanghai, China). Paclitaxel (PTX) and N-acetylcysteine (NAC) were purchased from Sigma-Aldrich Corp. (St. Louis, USA). SignalSilence® FOXO1 siRNA (6256) and Control siRNA (6568) were purchased from Cell Signaling Technology (Beverly, USA). Lipofectamine™ 2000 transfection reagent was purchased from Thermo Fisher Scientific, Inc. (Carlsbad, USA); BCA protein quantification kit (FD2001) was purchased from Fdbio Science (Hangzhou, China). Mammalian target of rapamycin (mTOR) (2983), phospho-mTOR (Ser2448) (5536), Akt (pan) (4691), phospho-Akt (Ser473) (4060), forkhead transcription factor (FOXO)-1 (C29H4) (2880), phospho-FOXO1 (Thr24) (4G6) (2599), caspase-3 (9662), caspase-9 (9508), B-cell lymphoma-extra large (Bcl-xL) (B9429), Bcl-2-associated X protein (Bax) (5023), and GAPDH (5174) primary antibodies were purchased from Cell Signaling Technology.

### 2.2. Constituents Analysis of XHP by GC-MS

The powered XHP (3.0g) were extracted with 70% ethanol at room temperature by ultrasonic extraction for 30 min. The extract solution was concentrated and then partitioned by 2 mL of hexane and 5 mL of water. The supernatant was transferred and evaporated to dryness. The oil extract was filtered by 0.22 *μ*M microporous membrane and stored at 4°C until analysis. The analysis was performed on an Agilent 6890/5973 GC-MS (Agilent Co., Palo Alto, CA, USA) using splitless injection mode, equipped with a TG-5MS capillary column (30 m × 0.25 mm × 0.25 *μ*m, Thermo Fischer Co., Waltham, MA, USA). Helium was used as carrier gas at a flow rate of 1.0 mL/min. The temperature of the split injector was 250°C and the split ratio was 10:1. 1mL sample solution was injected into GC system. The column was initiated at 80°C at a rate of 8°C/min to 200°C and kept for 15 min, then to 260°C at 10°C/min and kept for 32min. The spectrometer was operated in the EI-mode at 70 eV and the photomultiplier voltage energy at 1988V. The ion source temperature was 230°C and the quadrupole temperature was 150°C. Mass scan range from 30 to 550 amu, and the components were identified by comparing with the data bank mass spectra (NIST 11 and Wiley 275).

### 2.3. Cell Line and Cell Culture

Human glioblastoma U-87 MG cells were obtained from the American Type Culture Collection (Rockville, USA). Cells were maintained in Dulbecco's modified Eagle's medium (DMEM) (Gibco, New York, USA) supplemented with 10% fetal bovine serum (Gibco, New York, USA) at 37°C in a humidified atmosphere of 5% CO_2_. After reaching 80% confluence, cells were used for analysis. All treatments were performed using 3% FBS supplemented medium.

### 2.4. Cell Viability Assay

The cell viability was examined by CCK-8 assay according to the manufacturer's instructions. Cells were seeded at a density of 5 × 10^3^ per well in 96-well plates (Corning, New York, USA). After cultured overnight, cells were treated with various concentrations of XHP (7.5, 15 and 30 *μ*g/mL) and incubated for 24h. Then 10 *μ*L of CCK-8 reagent was added to the culture medium and incubated at 37°C for 1-2h. The absorbance was measured at a wavelength of 450 nm using a microplate reader (Thermo FC, Waltham, USA) to reflect cell viability. The % cell inhibition was determined using the following formula:

(1) %  cell  inhibition=1−ODsample−ODblankODcontrol−ODblank×100

Nonlinear regression graph was plotted between % cell inhibition and Log10 concentration and calculation of IC_50_ value was determined using GraphPad Prism software 5.0 (GraphPad, San Diego, CA). Each assay was repeated in triplicate and the results were given as mean ± SD of independent experiments.

### 2.5. Cell Cycle Assay by Flow Cytometry

Based on CCK-8 assay results, the same three concentrations of XHP (7.5, 15, and 30 *μ*g/mL) were used for the cell cycle assay. U-87 MG cells were collected after treatment for 24h and then fixed in 80% ethanol at 4°C overnight. On the next day, the cells were washed and suspended in phosphate-buffered saline (PBS) containing 50 *μ*g/mL propidium iodide at 37°C for 30min. The fluorescence-activated cells were further analyzed on a FACS Calibur (BD Biosciences, San Joe, USA).

### 2.6. Apoptosis Assay by Flow Cytometry

Apoptotic U-87 MG cells were detected by Annexin V-FITC/PI double staining with the apoptosis detection kit. Briefly, cells were harvested after XHP (7.5, 15, and 30 *μ*g/mL) treatment for 24h. Then, cells were centrifuged at 2000 rpm; the pellets were washed twice using PBS. Subsequently, cells were resuspended and labeled in the fluorochrome (5 *μ*L Annexin V and 10 *μ*L propidium iodide) and incubated for 15min at room temperature in darkness. After that, flow cytometry analysis was performed on a FACS Calibur. The fluorescence of FITC and PI were measured in the FL1 channel and FL2 channel (*λ*_ex_ = 488 nm, *λ*_em_ = 530 nm), respectively. 1 × 10^5^ events were recorded for each sample. The data were analyzed by FlowJo software V7.6 (ETree star, Ashland, OR).

### 2.7. Apoptosis Detection by TUNEL Staining

Apoptotic cells were detected using TUNEL FITC apoptosis detection kit. Briefly, after being treated with XHP (7.5, 15 and 30 *μ*g/mL) for 24h, U-87 MG cells were fixed with 4% Paraformaldehyde (PFA), permeabilized with 0.2% Triton X-100 and labeled with TdT reaction mix. DAPI (4′, 6-diamidino-2-phenylindole) was used to stain nuclei. Confocal microscopy (Nikon C2plus, Tokyo, Japan) was applied to observe morphological nuclear DNA fragmentation in the stained U-87 MG cells.

### 2.8. Measurement of Mitochondrial Membrane Potential (ΔΨm)

Mitochondrial membrane potential (MMP) was measured by a JC-1 assay kit. U-87 MG cells were seeded in 6-well plates (2 × 10^5^/well) and incubated at 37°C overnight. Next, the cells were harvested after the desired treatments with XHP for 24h and then incubated in darkness at 37°C for 30min with 10 mM JC-1 contained PBS buffer. Finally, The green (JC-1 monomers) and red (JC-1 aggregates) fluorescence ratio that measured the proportion of mitochondrial depolarization was acquired on a FACS Calibur.

### 2.9. Detection of Intracellular ROS Levels by Flow Cytometry

Intracellular ROS production was detected using reactive oxygen species assay kit according to the manufacturer's instruction. After U-87 MG cells pretreated with 30 *μ*g/mL XHP for 0.5h, 1h, or 3h, the cells were loaded with fluorescent probe DCFH-DA at a final concentration of 10 *μ*M in serum-free cell culture medium for 30 min at 37°C in the dark. After DCFH internalization, the cells were washed three times with serum-free cell culture medium to remove excess DCFH-DA, then the cells were collected and the fluorescence intensity of DCF was quantified on a FACS Calibur (*λ*_ex/em_ = 488/525 nm). 100 mM H_2_O_2_ was used as a positive control.

### 2.10. Western Blot Analysis

After being treated with XHP for 24h, U-87 MG cells were lysed with a NP-40 buffer and the total protein was extracted using radio immunoprecipitation assay buffer containing 1% protease inhibitor cocktail (Roche, Basel, Switzerland). The protein concentration was measured using a BCA protein assay kit (Fdbioscience, China). 20-50 *μ*g of cellular proteins was electroblotted onto PVDF membranes following separation on 12% SDS-PAGE gel electrophoresis. Membranes were blocked with 5% no-fat milk at room temperature for 1h and then blotted with primary antibodies (Bcl-xL, Bax, caspase-3, caspase-9, Akt, p-Akt, mTOR, p-mTOR, FOXO1, p-FOXO1, and GAPDH) at 4°C overnight with a 1:1000 dilution. Blots were then incubated with HRP-conjugated secondary antibodies at room temperature for 1h, followed by ECL development (Bio-Rad Laboratories, Hercules, USA). The images were captured and documented with a CCD system (imagestation 2000 MM, Kodak, Rochester, USA) and gray density was analyzed using ImageJ software (NIH, USA). Protein expression levels were determined using GAPDH as an internal control.

### 2.11. Immunofluorescence Staining

After being treated with XHP for 24h, U-87 MG cells were fixed with 4% paraformaldehyde for 10min followed by washing with PBS. Then cells were immunostained with anti-FOXO1 or anti-p-FOXO1 antibody (1:100) at 4°C overnight and stained with Alexa Fluor 568-conjugated secondary IgG (1:500, Life Technologies, Carlsbad, CA, USA) for 1h at room temperature. Cells were incubated with DAPI to stain nuclei, and images were acquired with a laser scanning confocal microscope (Olympus FV1000, Japan).

### 2.12. Transient Transfection with siRNA

When reaching 70% confluence, the U-87 MG cells were transfected with human specific FOXO1 siRNA or a control siRNA at 10 nM by using Lipofectamine 2000 according to the manufacturer's instruction. After being incubated with siRNA for 8h, cells were washed thrice to remove transfection reagents and then were treated with XHP and incubated for another 24h, followed by cell viability assessment using CCK-8 assay. Knockdown of FOXO1 was confirmed by western blot analysis and Q-PCR.

### 2.13. Quantitative RT-PCR Analysis

Total RNA was extracted from treated U-87 MG cells using Trizol reagent (Invitrogen, Carlsbad, USA) and was reverse-transcribed into cDNA using a SuperScript™ III First-Strand Synthesis SuperMix (Invitrogen, Carlsbad, USA). The primers' sequences were as follows: FOXO1 (FP: 5′-TCGTCATAATCTGTCCCTACACA-3′; RP: 5′-CGGCTTCGGCTCTTAGCAAA-3′). Quantitative RT-PCR was performed using SYBR green supermix (Bio-Rad, Hercules, CA, USA) with GAPDH used as endogenous controls in a MX3005P multiplex quantitative qPCR system (Stratagene, California, USA).

### 2.14. Statistical Analysis

Statistical analysis was performed using GraphPad Prism software 5.0. All data were presented as mean value ± SD. The significance of difference between groups was assessed using Student's* t*-test. *P* values < 0.05 were considered to be statistically significant.

## 3. Results

### 3.1. Analysis of Compounds in XHP by GC-MS

**Figure 1:**
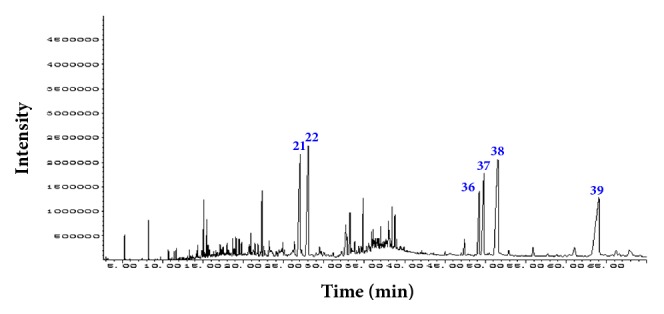
*The total ion chromatogram of XHP obtained from GC-MS analysis*.

To get more acquaintance about the active ingredients involved in XHP, GC-MS analysis was applied. It is revealed that total thirty-nine chromatographic peaks were separated well and thirty-two constituents were identified from them (the identified chemical compounds were presented in Table S1). Six compounds, 24-norursa-3,12-diene (17.72%), 24-norursa-3,12-dien-11-one (16.28%), 3,14,15-trihydroxypregn-16-en-20-one (11.77%), isopropyl-1,5,9-trimethyl-15-oxabicyclo[10.2.1] pentadeca-5,9-dien-2-ol (11.40%), 24-noroleana-3,12-diene (8.45%), and 24-norursa-3,9(11),12-triene (5.98%), corresponding to peaks 38, 39, 21, 22, 37, and 36 in the chromatographic spectrum, comprised 71.60% of total amount and could be regarded as the major constituents of XHP (Figure 1).

### 3.2. XHP Inhibited U-87 MG Cell Growth

**Figure 2:**
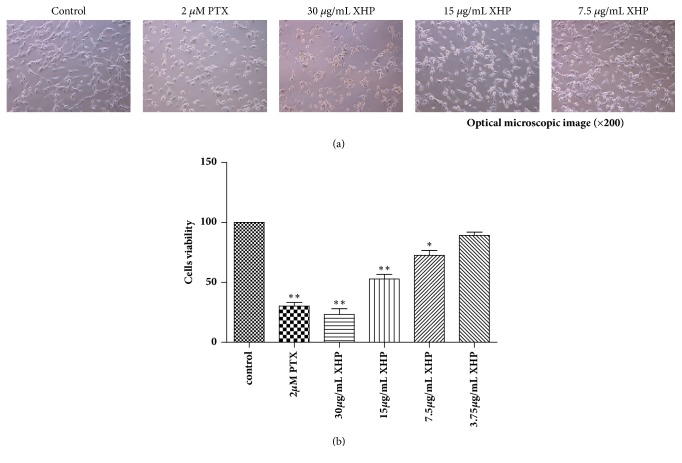
*Inhibitory effects of XHP on the growth of U-87 MG cells*. U-87 MG cells were treated with the indicated concentrations of XHP or PTX for 24h, (a) Morphological changes of cell confluence were observed under phase-contrast microscopy (× 200). (b) Cell viability was determined by CCK-8 assay. IC_50_ of PTX on U-87 MG cells was 4.67 ± 0.07 *µ*M after 24h treatment (n = 3, mean ± SD). *∗P *< 0.05, *∗∗P *< 0.01 significantly different from the control.* P* values were determined by Student's* t*-test.

To determine the effect of XHP on U-87 MG cell growth, cells were treated with different concentrations of XHP for 24h and CCK-8 assay was performed. It could be found that cell densities were decreased obviously in a dose-dependent manner. XHP-induced cell morphological changes and decreased the viable cell number observed under optical microscope. The IC_50_ of XHP on U-87 MG cell was 14.60 ± 1.94 *μ*g/mL after 24h treatment ( Figures 2(a) and 2(b)).

### 3.3. XHP Arrested Cell Cycle of U-87 MG Cell

**Figure 3:**
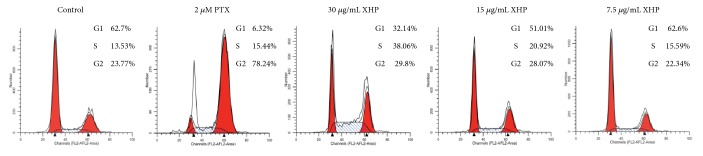
*Cell cycle analysis on U-87 MG cells by FACS*. XHP-induced cell cycle arrest in S-phase in U87 MG cells in a dose-dependent manner.

FACS analysis was conducted to assess the effects of XHP on cell cycle progression. The results showed that after being treated with XHP at 7.5, 15 or 30 *μ*g/mL, the cell accumulation percentage in S-phase was at 16.71 ± 2.18%, 21.32 ± 2.09% (*P* < 0.05, n = 3) and 38.76 ± 1.95% (*P* < 0.01, n = 3), compared with 13.53 ± 1.83% in the control group. The cell decreased percentage in G_0_/G_1_ phase was at 60.17 ± 1.08%, 51.19 ± 0.53% (*P* < 0.05, n = 3), and 39.33 ± 0.97% (*P* < 0.01, n = 3), compared with 63.73 ± 1.16% in the control group (Figure 3). No obvious change was observed in the G_2_/M phase. The results indicated that XHP could arrest U-87 MG cells in S-phase cell cycle and subsequently block cell growth.

### 3.4. XHP-Induced Apoptosis of U-87 MG Cells

**Figure 4:**
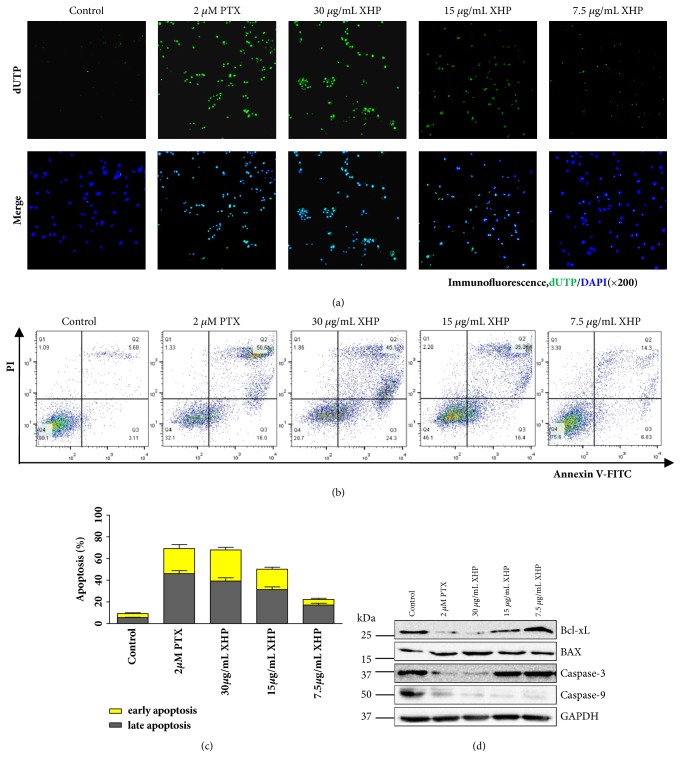
*XHP-induced apoptosis in U-87 MG cells. *(a) Confocal images of U-87 MG cells treated with different concentrations of XHP determined by TUNEL assay stained by FITC-dUTP and DAPI (× 200). (b) Effects of XHP on cell apoptosis in U-87 MG cells after 24h treatment determined by FACS following AnnexinV-FITC and propidium iodide double staining. The fluorescence of FITC and PI were measured in the FL1 channel and FL2 channel, respectively. A total of 10^5^ events were recorded for each sample before any gate setting and were analyzed with the FlowJo software V7.6. Cells in the right lower quadrant are undergoing early apoptosis. Cells in the right upper quadrant are undergoing late apoptosis. PTX was used as a positive control. Representative results are shown (n = 3). (c) U-87 MG cells were incubated with different concentrations of XHP, 2 *µ*M PTX, or control medium for 24h, stained with AnnexinV-FITC and propidium iodide and subjected to flow cytometry analysis. Quantified data of apoptosis cells after treatments with varying formulations (n = 3, mean ± SD). (d) After being incubated with different doses of XHP, 2 *µ*M PTX, or control medium for 24h, U-87 MG cells were harvested and subjected to western blot analysis with anti-Bcl-xL, anti-Bax, anti-caspase-3, and anti-caspase-9 antibodies.

TUNEL, FACS, and western blot assay were used to detect the apoptosis induced by XHP on U-87 MG cells. As shown in Figure 4(a), the increased DNA fragmentation accompanied with the increase of XHP concentrations was observed after the treatment for 24h with FITC-dUTP and DAPI double staining in the TUNEL assay. Moreover, the proapoptotic ability of XHP was evaluated by FACS. We found that the apoptotic ratio was increased in the XHP-treated group versus the control group in a concentration-dependent manner (Figures 4(b) and 4(c)), and both of the early-period and late-period apoptotic percentage were improved. In particular, the percentage of early-period apoptotic cells with 30 *μ*g/mL of XHP was significantly higher than the untreated control cells from 3.11% to 24.30% (*p* < 0.01). To confirm the association between XHP treatment and apoptosis, the effects of XHP on the modulation of apoptosis-related proteins were evaluated by western blot analysis. As shown in Figure 4(d), the proapoptotic protein Bax was upregulated and the antiapoptotic protein Bcl-xL was downregulated in a dose-dependent manner. In other words, Bax/Bcl-xL ratio was increased by XHP treatment. In addition, their downstream proteins procaspase-3 and procaspase-9 were also activated by XHP. These findings suggested that XHP might induce apoptosis of U-87 MG cells by upregulating the expression of proapoptotic protein Bax and then subsequently activate its downstream proteins caspase-9 and caspase-3. From results above, it indicated that XHP could decrease the viability of U-87 MG cells, induce cell apoptotic death, and affect apoptotic associated proteins expression.

### 3.5. XHP-Induced Apoptosis via the ROS-Mediated Mitochondrial-Dependent Pathway

**Figure 5:**
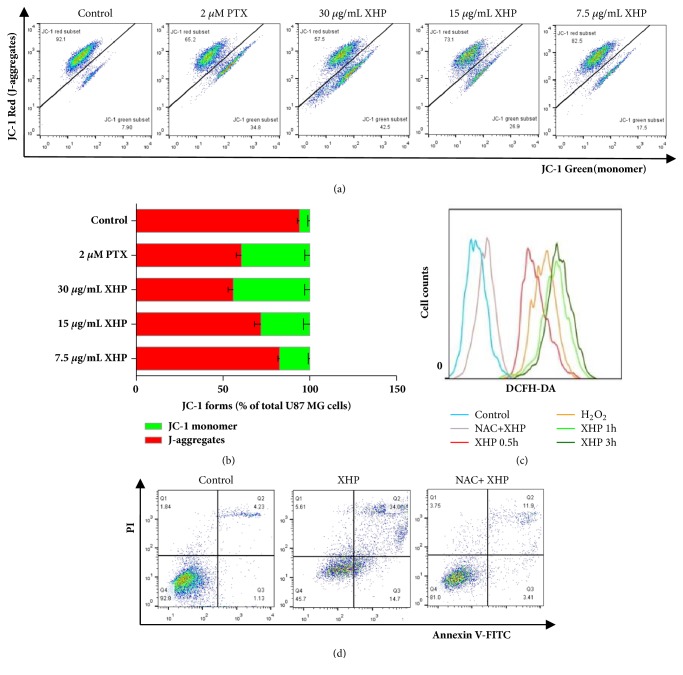
*ROS generation and mitochondrial dysfunction were required for XHP-induced U-87 MG cells apoptosis*. (a) 2 × 10^5^ U-87 MG cells were treated with PTX or XHP for 24h and incubated with 10 mM JC-1 in darkness at 37°C for 30min, then ΔΨm was measured with FACS. Detection wavelengths were 530 nm for the green fluorescence and 585 nm for the red fluorescence. (b) Quantified data in JC-1 absorbed cells (n = 3, mean ± SD). (c) Cells were pretreated with or without ROS inhibitors (5 mM NAC) for 30 min, then were exposed to 30 *µ*g/mL XHP for 0.5h, 1h or 3h, and then were collected and stained with DCFH-DA for measurement of H_2_O_2_ by FACS. H_2_O_2_ (100 mM) was used as positive control. (d) Cells were pretreated with ROS inhibitors (5 mM NAC) for 30min and then treated with the indicated procedure. FACS was employed to evaluate apoptosis. Each experiment was performed in triplicate.

Collapse of mitochondrial membrane potential (ΔΨm) was regarded as a key factor of the mitochondrial-dependent apoptotic pathway. The changes of ΔΨm were detected by JC-1 analysis. As shown in Figures 5(a) and 5(b), the accumulation of monomer improved remarkably in the XHP-treated cells, and J-aggregates decreased at the same time, which suggested that XHP could affect early apoptosis of U-87 MG cells. Furthermore, to investigate whether ROS was involved in the mitochondrial damage, the intracellular H_2_O_2_ level was detected by the fluorescent probe DCFH. As shown in Figure 5(c), XHP triggered intracellular ROS release significantly. After using the ROS scavenger NAC, the ROS generation, cell apoptosis, and the oxidative stress induced by XHP were all reversed (Figures 5(c) and 5(d)). Taken together with the above findings, the results suggested that XHP-induced apoptosis might be related to the ROS-mediated mitochondrial-dependent pathway.

### 3.6. XHP Activated the Akt/mTOR/FOXO1 Signaling Pathway

**Figure 6:**
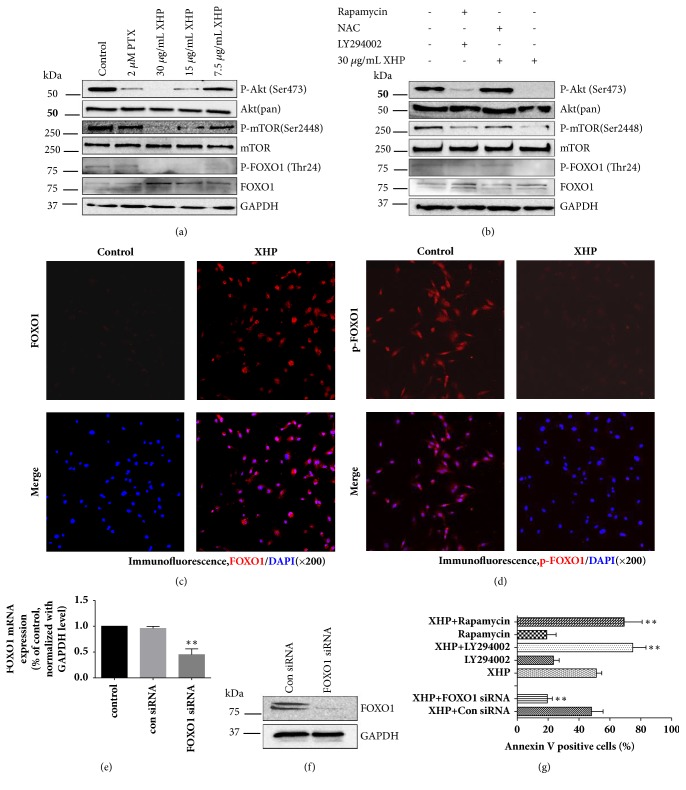
*The Akt/mTOR/FOXO1 signaling pathway was associated with the antigliomas of XHP. * (a) After being incubated with different doses of XHP, 2 *µ*M PTX or control medium for 24h, U-87 MG cells were lysed and subjected to western blot analysis of Akt, p-Akt, mTOR, p-mTOR, FOXO1, and p-FOXO1 antibodies. (b) Western blot analysis of AKT, p-Akt, mTOR, p-mTOR, FOXO1, and p-FOXO1 expression in U-87 MG cells after treatment with LY294002 (20 *μ*M), Rapamycin (50 nM), or NAC (5 mM) with or without XHP. (c) Confocal images of U-87 MG cells treated with XHP stained with anti-FOXO1 antibody. (d) Confocal images of U-87 MG cells treated with XHP were stained with anti-p-FOXO1 antibody. (e) Quantitative RT-PCR analysis of U-87 MG cells subjected to FOXO1-specific knockdown, results were normalized with GAPDH level and expressed as folds of control (n = 3, mean ± SD). (f) U-87 MG cells subjected to FOXO1-specific knockdown were assayed by western blot. (g) U-87 MG cells treated with XHP in the absence or presence of LY294002 (20 *μ*M), Rapamycin (50 nM), or FOXO1-specific siRNA (10 nM). The proportion of Annexin V positive cells was measured by flow cytometry. Data are mean ± SD of three different experiments.*∗P* < 0.05, *∗∗P *< 0.01 significantly different from the control.

Accumulating evidence suggested that Akt, mTOR, and FOXO1 played important roles in the proliferation of U-87 MG cells. To investigate the mechanism of the apoptosis induced by XHP, Akt/mTOR/FOXO1 signaling pathway deserved to be evaluated. As shown in Figure 6(a), the phosphorylation of Akt, mTOR, and FOXO1 was downregulated after treatment with different concentrations of XHP, while FOXO1 expression was significantly increased under the same manner. As a transcription factor, the nuclear localization of FOXO1 defined its role in the regulation of downstream targets. In this experiment, the distribution of FOXO1 between nuclear and cytosolic compartments was studied by immunofluorescent imaging. It was observed that FOXO1 accumulation and p-FOXO1 reduction in nuclei were synchronous in XHP-stressed U-87 MG cells (Figures 6(c) and 6(d)). Besides, it was also found that the proapoptotic effect of XHP on Akt/mTOR/FOXO1 signaling pathway could be reversed by NAC (Figure 6(b)). Moreover, Akt inhibitor LY294002, mTOR inhibitor rapamycin, and FOXO1-specific siRNA were adopted to verify the roles of Akt, mTOR, and FOXO1 in the apoptotic process. After being treated with LY294002 or rapamycin, the sensitivity of U-87 MG cells to XHP and the apoptotic cells were all increased, yet the inactivation of FOXO1 by siRNA attenuated the sensitivity of U-87 MG cells to XHP (Figure 6(g)). Consequently, it is suggested that XHP could inhibit the Akt/mTOR/FOXO1 pathway to exert its antiglioblastoma effect.

## 4. Discussion

XHP, a representative traditional Chinese medicine formula for clearing heat and relieving toxin, shows remarkable antineoplastic properties against several cancers including glioma in clinical practice. However, its pharmacological activity and underlying mechanism on glioblastoma have not been well studied until now. Herein, human U-87 MG cells were used as an experimental model to examine its antiglioma effects. Our findings preliminary demonstrated that XHP could inhibit U-87 MG cells proliferation and induce apoptosis via mitochondrial damage and inhibition of ROS-mediated Akt/mTOR/FOXO1 signal pathway.

Apoptosis is a vital mechanism to balance cell proliferation and death. In our study, after being treated with XHP, apoptosis-related morphology changes in cells, such as cell shrinkage, chromatic agglutination, and nuclear fragmentation, were observed. Many reports showed that apoptosis often occurred with cell cycle arrest in G_1_ or G_2_/M phase via interfering with the particular cyclins [23, 24]. However, cells were found to be blocked from G_1_ to S-phase in our study. This result was in agreement with the previous reports on XHP treatments in human hepatocellular carcinoma BEL-7404 cells and human ovary carcinoma Sk-OV-3 cells [25, 26]. It could be presumed that XHP might impact DNA synthesis and/or a certain cyclin that regulated S-phase [27, 28].

Bcl-2 family proteins regulate mitochondria-dependent apoptosis by controlling mitochondrial permeability and the release of cytochrome c (Cyt* c*) [29, 30], with the balance of anti- and proapoptotic members arbitrating cell fate. Antiapoptotic proteins Bcl-2 and Bcl-xL reside in the outer mitochondrial wall and inhibit Cyt* c* release. Proapoptotic Bcl-2 proteins such as Bad (Bcl-xL/Bcl-2 associated death promoter), Bid (BH3 interacting agonist), Bax and Bim (Bcl-2 interacting mediator of cell death) reside in the cytosol but translocate to mitochondria following death signaling, where they could promote the release of Cyt* c*. Activated Bad, an essential initiator of the apoptotic cascade, translocates to mitochondria and forms a proapoptotic complex with the antiapoptotic mitochondrial proteins Bcl-2 and Bcl-xL, as well as to antagonize their antiapoptotic activity and promote the proapoptotic activity of Bax [31–33]. Bax and Bim translocate to mitochondria in response to death stimuli including survival factor withdrawal, activated following DNA damage, inducing transcription of Bax. Bax translocates to the outer mitochondrial membrane through BH3 and multimerizates, which forms membrane channels to stimulate the release of mitochondrial Cyt* c* and apoptosis-inducing factor (AIF). Released Cyt* c* binds apoptotic protease-activating factor 1 (Apaf1) and forms activated apoptotic bodies with caspase-9, leading to a cascade of downstream caspase reaction [34, 35]. Although the mechanism(s) regulating mitochondrial permeability and the release of Cyt* c* during apoptosis are not totally understood, Bcl-xL and Bax apparently influence the release of Cyt* c* [33, 36]. In this study, we observed that XHP-induced apoptosis in U-87 MG cells from Annexin V/PI staining and DNA fragmentation (Figures 4 and 5). The appeared mitochondrial dysfunction detected by JC-1 assay confirmed that the U-87 MG cell apoptosis occurred via mitochondria-dependent pathway. In addition, oxidative imbalance was accompanied with the decreased mitochondrial membrane potential. As is well known, increased intracellular ROS level plays a critical role in mitochondria-dependent apoptotic pathway [37, 38]. Excessive ROS could cause dysfunction of the mitochondrial membrane proteins and collapse of mitochondrial membrane potential. Application of NAC, the ROS inhibitor, could usually alleviate oxidative stress and reduce ROS generation, as well as the ratio of apoptosis. In our study, after being pretreated with XHP, rapid ROS generation and relief by NAC were observed (Figure 5). It is implied that XHP-induced apoptosis was at least partly depending on ROS generation. Furthermore, it was found that XHP significantly alter Bax and Bcl-xL levels in U-87 MG cells, increase the Bax/Bcl-xL ratio, and decline of the precursor forms of caspase-9 and caspase-3. Further investigation might be needed to clarify whether XHP affects other Bcl-2 family members such as Bim, Bad, and BH3-only proteins in U-87 MG cells.

It is clear that the clinical significance of Akt and mTOR are crucial in GBM. Both of them can be used as markers to predict prognosis and target candidates for personalized medicine [39–42]. Their activation promotes cell proliferation, regulates cellular energy metabolism, and provides protection from apoptosis. PI3K/Akt/mTOR pathway emerges as a potential treatment for GBM patients. FOXOs, comprising FOXO1, FOXO3, FOXO4, and FOXO6, are the focus of cancer research recently [43, 44]. FOXOs have emerged as tumor suppressors via inhibiting PI3K/Akt signaling pathway, which can be phosphorylated at various serine and threonine residues (S253, S315 and Thr32) by activated Akt. Once phosphorylation accomplished, FOXOs are exported from nucleus to cytoplasm and become inactive, which leads to inhibition of transcriptional activity [45]. Therefore, nuclear localization of FOXOs is required for their transcriptional regulatory functions, such as cell proliferation, differentiation, cell cycle regulation, protection from oxidative stress, and apoptosis [46]. In this study, we attempted to investigate whether XHP exerted U-87 MG cells apoptosis by regulating the above-mentioned signaling pathways.

From western blot results, we found that XHP could inhibit the phosphorylation of the key protein members in PI3K/Akt pathway, including p-Akt (Ser473), p-mTOR (Ser2448), and p-FOXO1 (Thr24), and upregulate total FOXO1 expression in U-87 MG cells (Figure 6). This result coincided well with the enhancement in nuclear localization and the potentiated cytoplasmic degradation of FOXO1 observed in immunofluorescence images. Activation of FOXO1 subsequently led to transcriptional activation of genes controlling cell cycle arrest or cell apoptosis, such as proapoptotic proteins [47]. We verified in this study that their ability of permeabilizing mitochondrial membrane could result in activation of executioner caspases such as caspase-9, caspase-3, and pinnacle of cell death [48]. Moreover, we found that synergetic effects occurred when XHP was combined with either Akt or mTOR inhibitors. Inhibition of Akt or mTOR can increase the cytotoxic and apoptotic effects of XHP, while FOXO1 knockdown could rescue U-87 MG cells from XHP-induced apoptosis. From above, it was obvious that the PI3K pathway proteins including Akt, mTOR, and FOXO1 are involved in XHP-induced apoptosis followed by the enhanced downstream proapoptotic target FOXO1's nuclear import. The activation of the dephosphorylated FOXO1 potentiated proapoptotic protein Bax expression and oxidative stress generation, which induced mitochondria-mediated activation of apoptotic cascade. Therefore, based on literature and our preliminary results, we hypothesized that XHP might inhibit glioblastoma cells apoptosis resistance by inducing Akt and mTOR dephosphorylation, decreasing phosphorylation of FOXO1, causing FOXO1 nuclear transport and enhancing its transcriptional activity, and finally triggering U-87 MG cell apoptosis.

## 5. Conclusion

The present study provided a consideration that XHP regulated glioblastoma cell apoptosis through suppressing Akt/mTOR/FOXO1 signaling cascade. This research might be helpful in understanding the mechanisms related to XHP against GBM.

## Data Availability

All relevant data are included within the article.

## Supplementary Materials

Supplementary 1 Table S1: Compounds identified by GC-MS analysis.

Supplementary 2 Figure S1: The total ion chromatogram of XHP obtained from GC-MS analysis.
