# Supplementary material for: Xihuang Pill Induces Apoptosis of Human Glioblastoma U-87 MG Cells via Targeting ROS-Mediated Akt/mTOR/FOXO1 Pathway
Source: Evid Based Complement Alternat Med. 2018 Jun 26;2018:6049498. doi: 10.1155/2018/6049498 (PMC6038446; doi:10.1155/2018/6049498)
Supplement: Supplementary 1 — Table S1: Compounds identified by GC-MS analysis. [file 6049498.f1.docx]

**Figure S1**. The total ion chromatogram of XHP obtained from GC-MS analysis.
