# Supplementary material for: Xihuang Pill Induces Apoptosis of Human Glioblastoma U-87 MG Cells via Targeting ROS-Mediated Akt/mTOR/FOXO1 Pathway
Source: Evid Based Complement Alternat Med. 2018 Jun 26;2018:6049498. doi: 10.1155/2018/6049498 (PMC6038446; doi:10.1155/2018/6049498)
Supplement: Supplementary 2 — Figure S1: The total ion chromatogram of XHP obtained from GC-MS analysis. [file 6049498.f2.docx]

**Table S1.** Compounds identified by GC-MS analysis

| **Peak No.** | **t_R_/min** | **Name** | **formula** | **Area%** |
| --- | --- | --- | --- | --- |
|  | 5.263 | 1-Octanol | C_8_H_18_O | 0.47 |
|  | 8.215 | Octyl acetate | C_10_H_20_O_2_ | 0.66 |
|  | 11.673 | β-Elemene | C_15_H_24_ | 0.20 |
|  | 14.323 | Lauric acid | C_12_H_24_O_2_ | 0.40 |
|  | 15.068 | Epicurzerenone | C_15_H_18_O_2_ | 1.06 |
|  | 15.446 | α-Copaene-8-ol | C_15_H_24_O | 0.70 |
|  | 15.613 | α-Cadinol | C_15_H_26_O | 0.22 |
|  | 17.075 | Procerin | C_15_H_18_O_2_ | 0.07 |
|  | 17.984 | Name: Isofuranodienone | C_15_H_18_O_2_ | 0.26 |
|  | 18.689 | Muscone | C_16_H_30_O | 0.57 |
|  | 19.072 | Citromycin | C_13_H_10_O_5_ | 0.49 |
|  | 19.109 | Unidentified |  | 0.38 |
|  | 19.473 | Myrrhanolide C | C_15_H_18_O_4_ | 0.43 |
|  | 19.765 | Methyl palmitate | C_17_H_34_O_2_ | 0.38 |
|  | 20.735 | Palmitic acid | C_16_H_32_O_2_ | 0.83 |
|  | 20.920 | Neocembrene A | C_20_H_32_ | 0.72 |
|  | 21.443 | 2-Heptyl-4-quinolinol 1-oxide | C_16_H_21_NO_2_ | 0.47 |
|  | 21.814 | 4-Acetyl-3-amino-5-ethyl-2-cyclopentene-1,1,2-tricarbonitrile | C_12_H_12_N_4_O | 0.43 |
|  | 22.317 | (S,E)-8,12,15,15-Tetramethyl-4-methylenebicyclo[9.3.1]pentadeca-7,11-diene | C_20_H_32_ | 2.84 |
|  | 23.208 | trans-5-Amino-5,6-dihydrochrysen-6-ol | C_18_H_15_NO | 0.65 |
|  | 27.044 | Isopropyl-1,5,9-trimethyl-15-oxabicyclo[10.2.1]pentadeca-5,9-dien-2-ol | C_20_H_34_O_2_ | 11.40 |
|  | 28.042 | 3,14,15-Trihydroxypregn-16-en-20-one | C_21_H_32_O_4_ | 11.77 |
|  | 32.707 | 4.Incensole oxide | C_20_H_34_O_3_ | 2.74 |
|  | 32.831 | Z-5-Methyl-6-heneicosen-11-one | C_22_H_42_O | 0.96 |
|  | 33.220 | 7,11-Dihydroxypregnane-3,20-dione | C_21_H_32_O_4_ | 2.47 |
|  | 33.797 | Unidentified |  | 0.60 |
|  | 34.851 | Spiro[7-acetoxy-2-hydroxy-2,4b-dimethylphenanthrene]-1,2'-(tetrahydropyran-5'-one) | C_21_H_32_O_5_ | 2.67 |
|  | 35.910 | Unidentified |  | 0.73 |
|  | 36.071 | Unidentified |  | 0.48 |
|  | 37.045 | Unidentified |  | 0.57 |
|  | 37.986 | 3-Ethyl-3-hydroxy-androstan-17-one | C_21_H_34_O_2_ | 0.94 |
|  | 38.049 | Unidentified |  | 0.72 |
|  | 38.440 | Unidentified |  | 1.21 |
|  | 38.772 | Lupeol | C_30_H_50_O | 1.09 |
|  | 47.399 | 3-(3,4-dimethylphenyl)-3-methyl-17-androstanone | C_28_H_40_O | 1.01 |
|  | 49.230 | 24-Norursa-3,9(11),12-triene | C_29_H_44_ | 5.98 |
|  | 49.809 | 24-Noroleana-3,12-diene | C_29_H_46_ | 8.45 |
|  | 51.543 | 24-Norursa-3,12-diene | C_29_H_46_ | 17.72 |
|  | 64.079 | 24-Norursa-3,12-dien-11-one | C_29_H_44_O | 16.28 |
